# Inositol hexaphosphate sensitizes hepatocellular carcinoma to oxaliplatin relating inhibition of CCN2-LRP6-β-catenin-ABCG1 signaling pathway

**DOI:** 10.7150/jca.62141

**Published:** 2021-08-24

**Authors:** Xia Liao, Yaoyao Zhang, Binghui Xu, Arshad Ali, Xin Liu, Qingan Jia

**Affiliations:** 1 Department of Nutrition, First Affiliated Hospital of Xi'an Jiaotong University, Xi'an 710061, China.; 2 Institute of Medical Research, Northwestern Polytechnical University, Xi'an, 710072, China.; 3 Department of Epidemiology and Biostatistics, School of Public Health, Xi'an Jiaotong University Health Science Center, Xi'an, 710061, China.

**Keywords:** Hepatocellular carcinoma, inositol hexaphosphate, oxaliplatin, Wnt signaling, CCN2

## Abstract

Hepatocellular carcinoma (HCC) is a drastic problem in China. Oxaliplatin, a platinum-based chemotherapy drug, has limited efficacy in treating HCC, characterized by intrinsic and acquired resistance. Inositol hexaphosphate (IP6), a carbohydrate abundant in grains, has contributed to the rising popularity of whole grain products consumption for the potential protection against dozens of diseases. However, the therapeutic potential of IP6 in halting the progression of HCC remains unclear, especially in combination with oxaliplatin. The anti-proliferation and anti-migration effects of IP6 were evaluated *in vitro* and *in vivo*. The synergistic and sequential anti-proliferative effect with IP6 and oxaliplatin were also evaluated in HCC. Finally, the role of CCN2-LRP6-β-catenin-ABCG1 signaling in oxaliplatin resistance and IP6 treatment was evaluated. We proved that IP6 treatment exhibited independent anticancer effect and synergistic anti-proliferative effects in combination with oxaliplatin in HCC. Specifically, up-regulation of ABCG1 and CCN2 were associated with oxaliplatin resistance. ABCG1 was acting as a downstream molecule of the CCN2-LRP6-Wnt/β-catenin signaling pathway in HCC cells. The IP6 treatment exhibited inhibition of CCN2-LRP6-Wnt/β-catenin signaling pathway and downregulation of ABCG1 in HCC cells. When combined with ABCG1 knocking down in HCC cells, the anti-proliferative effect of oxaliplatin was partly impaired in combination with IP6. We suggested that IP6 treatment renders HCC sensitive to oxaliplatin and breaking the CCN2-LRP6-β-catenin-ABCG1 signaling pathway is one of the mechanism after IP6 treatment.

## Introduction

Liver cancer, most commonly seen as hepatocellular carcinoma (HCC), has high prevalence and incidence rates in China, which accounts for more than 50% of the total number of liver cancer cases and deaths in the world 1. One of the chemotherapeutic drugs for patients with advanced HCC is oxaliplatin, which initiates apoptosis by inhibiting the replication and transcription of DNA in HCC cells 2. However, the efficacy of oxaliplatin on HCC is poor, exhibiting intrinsic and acquired resistance.

Traditional Chinese medicine (TCM) has been demonstrated to be effective for the treatment and improving drug resistance of HCC 3. Whole grains are widely recommended as an integral part of healthy diets, especially in malignant tumor patient. In addition, evidence from previous studies have shown that grains may protect against various cancers 4. Inositol hexaphosphate (IP6) is a naturally occurring polyphosphorylated carbohydrate that is abundant in grains and is shown to have broad-spectrum biological functions 5, 6. Preliminary study has shown that IP6 could be a novel treatment for a wide variety of tumor 7. However, the therapeutic potential and related molecular mechanism of IP6 in halting the progression of HCC remains unclear, especially in oxaliplatin resistance.

Previously, we generated an oxaliplatin-resistant HCC cell line and using cDNA microarrays found that the expression levels of 332 genes were significantly changed compared to control non-oxaliplatin-resistant HCC cells 8, including ATP-binding cassette family proteins and CCN family proteins. And we had also proved that CCN2 plays a promoting role in HCC progression and stemness maintenance through LRP6 9 and MAPK 10 signaling pathways. ABCG1 is a cholesterol lipid efflux pump that plays a well-known role in tumor growth, conferring chemoresistance to various malignant tumors 11 and presenting a major obstacle for effective clinical cancer treatment 12. The discovery of ABCG1 not only offered an explanation as to why treatment with oxaliplatin ultimately failed to eradicate the tumor, but it also had a profound impact on the current perception of drug resistance reversal 13. Up to now, the relationship between CCN2 and ABCG1 in the maintenance of HCC oxaliplatin resistance, and the role and mechanism of IP6 in reversing oxaliplatin resistance are still vague.

In this study, we confirmed that IP6 treatment inhibited proliferation and migration *in vitro* and *in vivo*. Then, we separately proved the enhanced anti-proliferative effects of combined and sequential treatment with IP6 and oxaliplatin in HCC. Finally, we proved that ABCG1 was acting as a downstream molecule of the CCN2-LRP6-Wnt/β-catenin signaling pathway in HCC, and IP6 treatment resulted in the downregulation of ABCG1 and inhibition of CCN2-LRP6-Wnt/β-catenin signaling, while ABCG1 was not the only target of IP6 treatment on HCC cells. This study suggests a strategy for using IP6 to reverse intrinsic and acquired chemotherapy resistance of HCC in the clinic.

## Materials and methods

### Cell lines and treatment procedures

Two human HCC cell lines were used in the study: MHCC97L, with high metastatic potential (established at Fudan University, Shanghai China), and Hep3B, with low metastatic potential (American Type Culture Collection, Rockville, MD, USA). All cells were maintained in Dulbecco's Modified Eagle's Medium (DMEM; GIBCO, Grand Island, NY), and supplemented with 10% fetal bovine serum (FBS; GIBCO) at 37 °C in a humidified incubator with 5% CO_2_. Cells were routinely screened for the presence of mycoplasma (Mycoplasma Detection Kit, Roche Diagnostics).

### Regents and antibodies

Oxaliplatin was purchased from ApexBio (Houston, TX, USA) and used for the generation of drug-resistant cell lines. Monoclonal antibodies were used for immunoblot analysis, including, β-catenin (Abcam, Cambridge, MA, USA), CCN2, p-LRP6, LRP6, β-actin, and ABCG1 (Proteintech, Chicago, USA). Water soluble potassium inositol hexaphosphate (IP6) was gifted from Ankang Shimao Biotech Company.

### Generation of IP6- and oxaliplatin-pretreated HCC cell lines

MHCC97L and Hep3B cells were seeded into T_25_ flasks and treated with 1 mM and 0.5 mM IP6 for 4 days, respectively. Then cells were passaged and a higher concentration of IP6 (1.5 mM and 0.75 mM) was added separately into MHCC97L and Hep3B cells. Finally, the cells were treated with final concentration of IP6 (2 mM and 1 mM), and the MHCC97L and Hep3B cells became stably resistant to IP6 at this concentration respectively. The cells were renamed MHCC97L-IP6 and Hep3B-IP6. Oxaliplatin-pretreated HCC cell lines were generated referring to the above method and renamed MHCC97L-OXA and Hep3B-OXA, and which was also described previously 8.

### Cell viability assay

HCC cells were seeded into 96-well plates and treated with oxaliplatin at increasing concentrations (0, 0.5, 1, 2, 4, 8, 16, 32, 64, 128 μM) for 24, 48, 72, and 96 h. Cell proliferation assays were carried out with the Cell Counting Kit 8 (CCK8; Dojindo Molecular Technologies, Inc.). Results were expressed as the absorbance of each well at 450 nm (OD450).

### Cell migration assays

Cell migration was assessed by transwell assays as described previously (Boyden chambers, Corning, Flintshire, UK) using MHCC97H and Hep3B 14. Then, 5 × 10^4^ cells in serum free DMEM with 1 mM IP6 were seeded into the upper chamber of each well on the membrane (8.0 μm pore size) of a 24-well plate. DMEM containing 10% fetal bovine serum (FBS) was added to the lower chamber of each well. After 48 h, cells reaching the underside of the membrane were stained with Giemsa (Sigma-Aldrich) and counted at × 200 magnification.

### Vector construction and lentivirus transduction

The human full-length cDNA of ABCG1 (NM_207630.1) were obtained from Genesent (Shanghai China) and then cloned into the pCDH. Using the In-Fusion HD Cloning Kit (Takara), the amplified fragment was inserted into the plasmid pCDH. Flag-tagged ABCG1 in pCDH vector was from Genesent (shanghai China). Lentiviral shRNA expression plasmids PLKO.1, three different shRNAs against ABCG1 mRNA were constructed: Sh1: TGGTGAGTTGGTGGCCATTAT; Sh2: AGGTGGTCTCGCTGATGAAAG; Sh3: TAGGAAGATGTAGGCAGATTG.

### Immunoblot and Immunohistochemistry analysis

The expression levels of CCN2, ABCG1, and Wnt signaling protein levels were determined by immunoblot assay according to the manufacturer's instructions. The total protein concentrations extracted from the cells were determined using the BCA protein assay kit (Beyotime Biotechnology, China). The following primary antibodies were used: anti-ABCG1 (1:1,000), anti-LRP6 (1:1,000) and anti-phospho-LRP6 (1:500), anti-β-catenin (1:1000), anti-CCN2 (1:1000), and β-actin (1:1000). Immunoblot reagents were purchased from Beyotime Biotechnology. Tumor tissue was fixed, embedded, and sliced into 5 μm thick sections. Immunohistochemical staining of CCN2 (1:300) and ABCG1 (1:500) was carried out using a standard protocol 15.

### Sphere Formation Assays

HCC cells (5 × 10^4^) were suspended in the appropriate amount of sphere-forming medium (DMEM + 15% FBS + 5% Matrigel matrix) in low-adhesion 6-well plates (Corning, New York, USA) for 3 weeks. Cells were treated with IP6 concentrations: 1 mM for MHCC97L and 0.5 mM for Hep3B cells. After 3 weeks, cells were photographed at 5 × magnification.

### *In vivo* evaluation of tumor proliferation and metastasis

MHCC97L cells were harvested, washed, and resuspended in PBS. Then 5 × 10^6^ cells (0.1 ml cell suspension) were injected into the left lobe livers of 4 weeks-old male BALB/c nu/nu mice. Each group contained 6 mice, and experimental group mice were treated orally with IP6 (2 mg /kg/d). Five weeks later, animals were sacrificed and subcutaneous tumor weights were evaluated. The numbers of lung metastatic nodules were evaluated by microscopy of serial sections of the right lung tissue block which is on behalf the entire pulmonary metastasis. Intraperitoneal injection of pentobarbital (5 mg/kg) combined with cervical spondylolisthesis was used for the killing of mice after the study. And this method was reported by our group in previous research 10. The study protocol was approved by the ethics committee on Experimental Animals of Northwestern Polytechnical University, and all methods were performed in accordance with the relevant guidelines.

### Statistical analysis

A two-sided Student's *t*-test was performed to evaluate statistical significance of differences in means. Experiments were performed at least three times and p < 0.05 was considered statistically significant. Statistical analysis was performed with SPSS 15.0 software for Windows (SPSS Inc. Chicago, IL, USA).

## Results

### IP6 directly inhibited the ability of proliferation and migration in HCC

**Figure 1:**
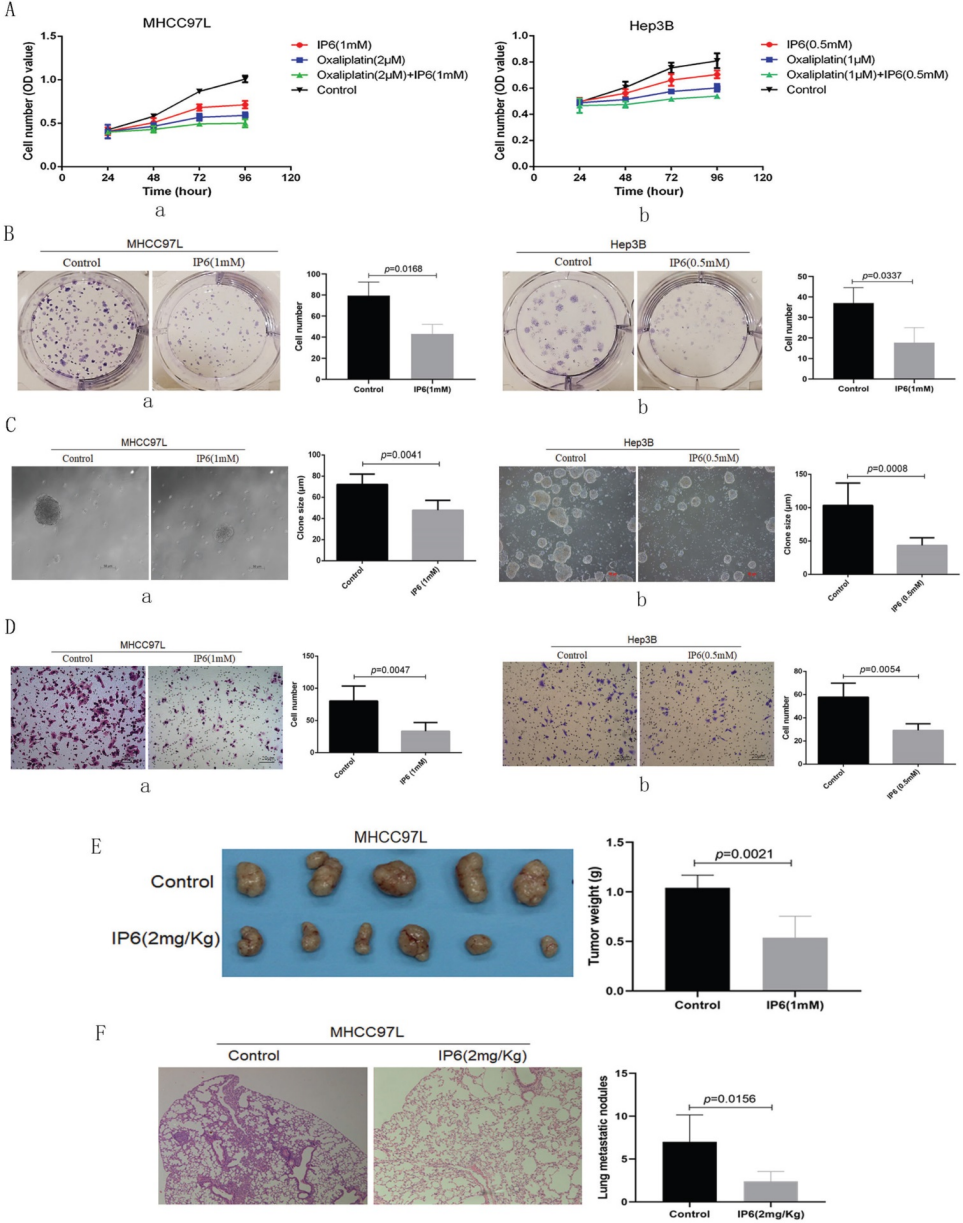
** IP6 directly inhibited the ability of proliferation and migration in HCC. (A)** Cell viability assays of MHCC97L and Hep3B cells treated with oxaliplatin and/or IP6 exhibited synergistically enhanced anti-tumor efficacy. **(B)** Adherent colony formation assays of MHCC97L and Hep3B cells treated with IP6 exhibited inhibited proliferation ability. **(C)** IP6 significantly inhibited sphere formation in MHCC97L and Hep3B cells. **(D)** The transwell assay demonstrated that MHCC97L and Hep3B cells migrated through the basement membrane less efficiently when treated with IP6. **(E)** IP6 attenuate ability of HCC proliferation with reduced subcutaneous tumor weights in IP6 group when compared to control group. **(F)** IP6 attenuate HCC pulmonary metastatic potential with reduced number of pulmonary nodules in IP6 group when compared to control group.

Cell viability was evaluated following individual treatment with IP6. The HCC cell lines MHCC97L and Hep3B exhibited lower proliferation rates following treatment with IP6 compared to control cells (Figure 1A, a, b). To confirm the action of IP6 in HCC proliferation using adherent clone, we treated MHCC97L (43.01 ± 9.17 *vs.* 79.33 ± 13.01; *p* = 0.0168; Figure 1B, a) and Hep3B (17.67 ± 7.37 *vs.* 37.13 ± 7.55; *p* = 0.0337; Figure 1B, b) cells with IP6, and we proved there was a significantly inhibited colony number. Then, sphere formation ability was examined through suspension culture using HCC cells treated with IP6. And we proved that IP6 significantly inhibited sphere formation in MHCC97L cells (72.83 ± 9.31 μm *vs.* 72.01 ± 9.87 μm; *p* = 0.0041; Figure 1C, a), and Hep3B cells (43.57 ± 11.44 μm *vs.* 103.41 ± 33.46 μm; *p* = 0.0008; Figure 1C, b). We also found IP6 significantly inhibited migration in MHCC97L (33.41 ± 13.69 *vs.* 80.01 ± 23.24; *p* = 0.0047; Figure 1D, a) and Hep3B (29.25 ± 5.62 *vs.* 57.75 ± 12.18; *p* = 0.0054; Figure 1D, b) cells.

To investigate whether IP6 could attenuate ability of HCC proliferation *in vivo*, subcutaneous xenografts were produced by administering subcutaneous injections of MHCC97L cells into the upper left flank region of the mice. Tumor weights were evaluated in five weeks after the treatments. And the tumor weight was reduced in IP6 group, when compared to control group (0.54 ± 0.22 g *vs.* 1.04 ± 0.13 g, *p* = 0.0021; Figure 1E). To investigate whether IP6 could attenuate HCC pulmonary metastatic potential, and the nodules of pulmonary metastasis were evaluated. And the number of pulmonary nodules was reduced in IP6 group, when compared to control group (2.41 ± 1.14 *vs.* 7.12 ± 3.16, *p* = 0.0156; Figure 1F). These findings suggested that IP6 treatment inhibited proliferation and migration in HCC.

### IP6 exhibited a synergistic and sequential anti-proliferative effect with oxaliplatin in HCC

**Figure 2:**
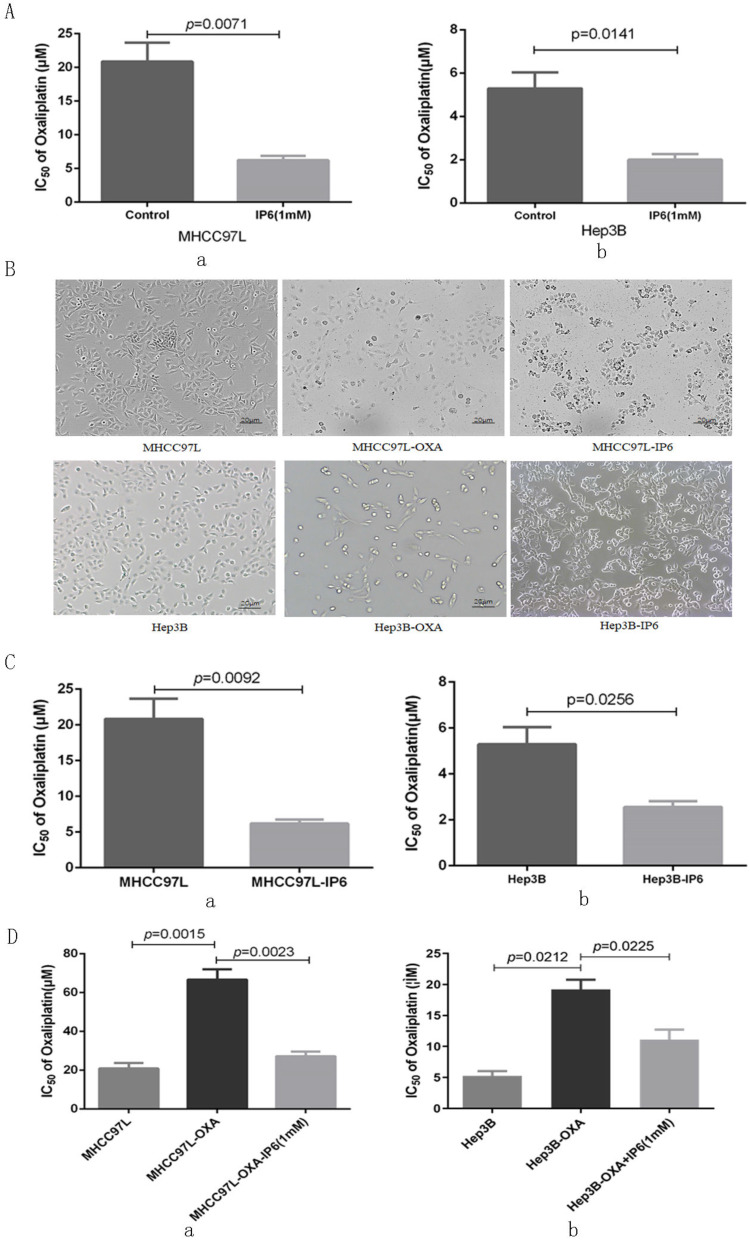
** IP6 exhibited a synergistic and sequential anti-proliferative effect with oxaliplatin. (A)** The response of HCC cells to combined therapy with different concentrations of oxaliplatin as assessed via measurement of half maximal inhibitory concentration (IC50) values, and IP6 also exhibited synergistically enhanced anti-tumor efficacy with lower IC50 values. **(B)** IP6-pretreated HCC cells exhibited increased intercellular adhesion and epithelioid cell morphology, which is contrary to the cell morphology from oxaliplatin-pretreated HCC cells. **(C)** IP6 pretreated HCC cells exhibited decreased oxaliplatin IC_50_ values. **(D)** The oxaliplatin-pretreated HCC cell lines were then treated with IP6, yielding significantly reduced resistance to oxaliplatin with decreased IC_50_ values in both MHCC97L-OXA and Hep3B-OXA.

Cell viability was evaluated following combination treatment with oxaliplatin and IP6. The MHCC97L and Hep3B HCC cell lines exhibited synergistically enhanced anti-tumor efficacy with combination treatment with the two drugs resulted in the lowest proliferation rate (Figure 1A). We next explored MHCC97L (6.22 ± 1.13 μmol/L* vs.* 20.85 ± 4.86 μmol/L; *p* = 0.0071; Figure 2A, a) and Hep3B (2.01 ± 0.46 μmol/L *vs.* 5.29 ± 1.29 μmol/L; *p* = 0.0141; Figure 2A, b) cells exhibited significantly decreased oxaliplatin IC_50_ values when treated in combination with IP6 compared to oxaliplatin treatment alone, thus indicating a synergistic anti-proliferative effect.

In order to simulate clinical sequential chemotherapy, MHCC97L and Hep3B cells were treated continually with IP6 to generate an IP6-pretreated cell line (MHCC97L-IP6, Hep3B-IP6) that exhibited lower proliferation rates with increased intercellular adhesion and epithelioid cell morphology. Meanwhile, we generated the oxaliplatin-pretreated cell line (MHCC97L-OXA, Hep3B-OXA), which exhibited lower proliferation rates with decreased intercellular adhesion (Figure 2B). MHCC97L-IP6 cells (6.19 ± 0.94 μmol/L *vs.* 20.85 ± 4.86 μmol/L; *p* = 0.0092; Figure 2C, a) and Hep3B-IP6 cells (2.56 ± 0.45 μmol/L *vs.* 5.29 ± 1.29 μmol/L; *p* = 0.0256) exhibited decreased oxaliplatin IC_50_ values compared to wide-type HCC cells (Figure 2C, b). MHCC97L-OXA cells (66.67 ± 9.02 μmol/L *vs.* 20.85 ± 4.86 μmol/L; *p* = 0.0015; Figure 2D, a) and Hep3B-OXA cells (19.61 ± 2.69 μmol/L *vs.* 5.29 ± 1.29 μmol/L; *p* = 0.0212; Figure 2D, b) exhibited increased oxaliplatin IC_50_ values compared to control cells. The oxaliplatin-pretreated HCC cell lines were then treated with IP6, yielding significantly reduced resistance to oxaliplatin with decreased IC_50_ values in both MHCC97L-OXA (27.18 ± 4.13 μmol/L with IP6 *vs.* 66.67 ± 9.02 μmol/L; *p* = 0.0023; Figure 2D, a) and Hep3B-OXA (11.09 ± 2.81 μmol/L with IP6 *vs.* 19.61 ± 2.69 μmol/L; *p* = 0.0225; Figure 2D, b) cells compared to the control groups. These findings suggested that IP6 reversed the tolerance of oxaliplatin-pretreated HCC with a synergistic and sequential anti-proliferative effect.

### The expression of ABCG1 and CCN2 in HCC cells was upregulated by oxaliplatin treatment and inhibited by IP6

**Figure 3:**
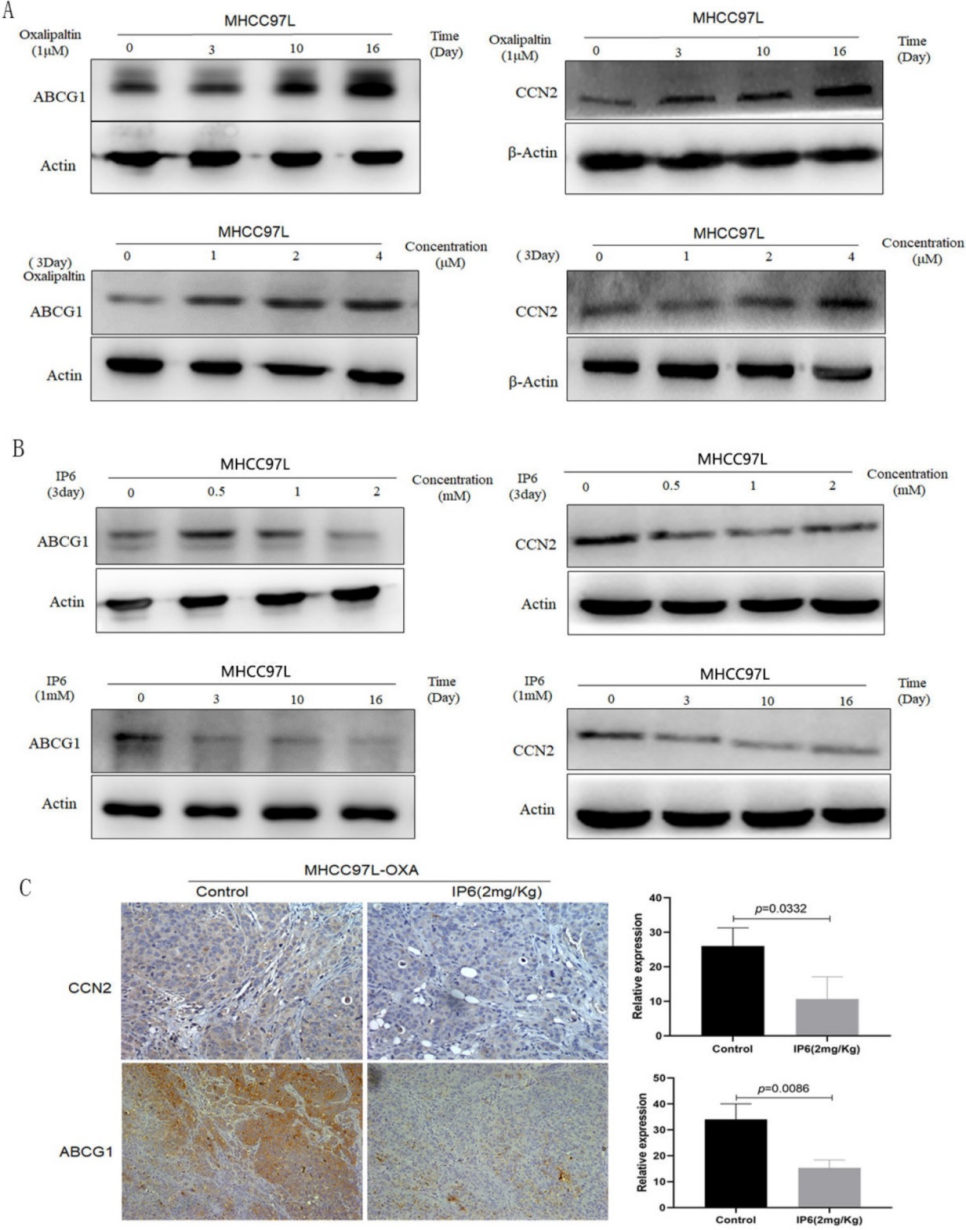
** ABCG1 and CCN2 expression was upregulated by oxaliplatin and significantly reversed by IP6. (A)** Immunoblot analysis verified that the expression of ABCG1 and CCN2 was significantly upregulated by oxaliplatin in a time- and concentration-dependent manner. **(B)** HCC cells treated with IP6 exhibited downregulated ABCG1 and CCN2 expression levels in a time- and concentration-dependent manner. **(C)** Tumor tissues from subcutaneous xenografts by immunohistochemistry exhibited a significantly decreased proportion of ABCG1 and CCN2 in IP6 treatment group.

Previously, we generated an oxaliplatin-resistant HCC cell line and found the expression levels of ATP-binding cassette family proteins and CCN family proteins were significantly changed compared to control non-oxaliplatin-resistant HCC cells, therefore, we focused on ABCG1 and CCN2 in this study 8. Immunoblot analysis verified that the expression of ABCG1 and CCN2 was significantly upregulated in response to oxaliplatin treatment in a time- and concentration-dependent manner. By treating MHCC97L cells with oxaliplatin using time and concentration gradients, we determined that the continuous treatment with 1 μM oxaliplatin for 16 days, or 4 μM oxaliplatin for 3 days, achieved the highest expression levels of ABCG1 and CCN2 (Figure 3A). Then MHCC97L cells were treated with IP6 using time and concentration gradients, and the expression of ABCG1 and CCN2 was downregulated as time or concentration increased (Figure 3B). We examined tumor tissues from subcutaneous xenografts by immunohistochemistry and also observed a decreased proportion of ABCG1 in IP6 treatment group (10.67 ± 6.43 *vs.* 26.12 ± 5.29, *p* = 0.0332; Figure 3C). Therefore, we confirmed that ABCG1 and CCN2 upregulation was involved in oxaliplatin resistance, which was significantly reversed by IP6.

### ABCG1 was a downstream molecule of the CCN2-LRP6-Wnt/β-catenin signaling pathway in HCC

**Figure 4:**
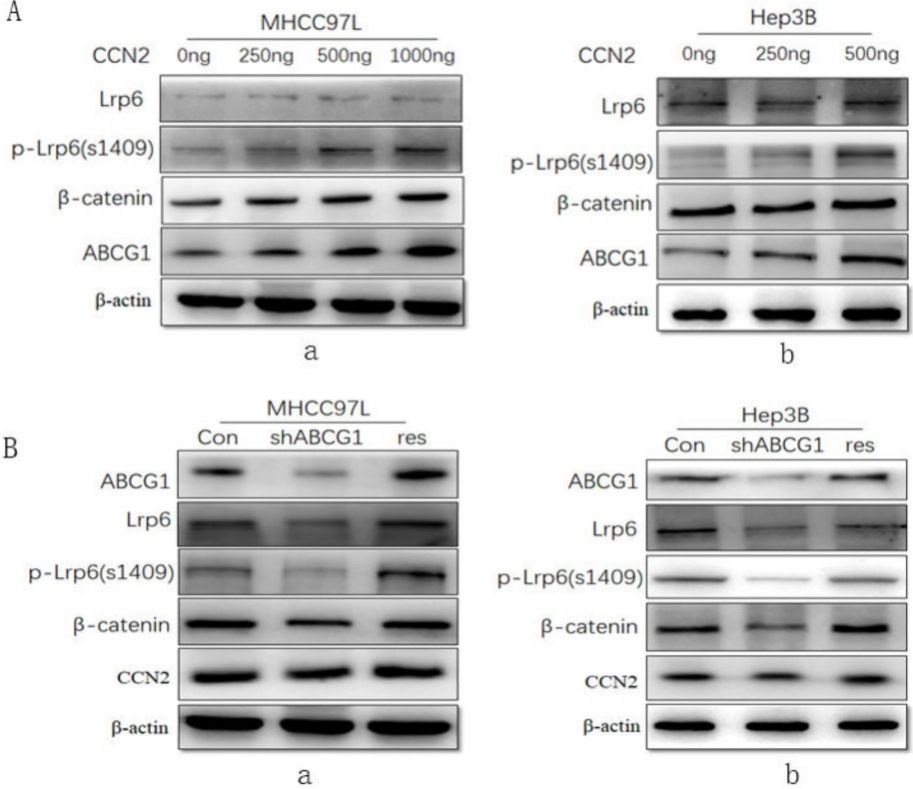
** ABCG1 was a downstream molecule of the CCN2-LRP6-Wnt/β-catenin signaling pathway in HCC. (A)** MHCC97L treated with recombinant CCN2 in increasing concentrations was able to activate Wnt signaling with concomitant upregulation of LRP6, phosphorylated LRP6, and β-catenin, and ABCG1 (a), and the trend was confirmed in Hep3B (b). **(B)** The downregulation of LRP6, phosphorylated LRP6, and β-catenin was proved after ABCG1 knocking down in HCC cell lines, and the trend was reversed following gene rescue of ABCG1 (a), and the same trend was also confirmed in Hep3B (b).

To investigate the effect of CCN2 on ABCG1 expression and the associated Wnt/β-catenin signaling pathway signaling, we treated MHCC97L cells with recombinant CCN2 in increasing concentrations, and found that CCN2 was able to activate Wnt signaling with concomitant upregulation of LRP6, phosphorylated LRP6, and β-catenin, and ABCG1(Figure 4A, a). And this trend was confirmed in another HCC cell line Hep3B after treated with CCN2 in increasing concentrations (Figure 4A, b).

Additionally, we investigate the effect of ABCG1 on CCN2 expression using RNA interference. After the expression of ABCG1 was knocked down, we found that the downregulation of LRP6, phosphorylated LRP6, and β-catenin, but the level of CCN2 exhibited no significant decrease in expression. Following gene rescue of ABCG1, the expression of LRP6, phosphorylated LRP6, and β-catenin was restored (Figure 4B, a). And this trend was also confirmed in another HCC cells Hep3B in the same way (Figure 4B, b). Therefore, we confirmed that CCN2 was the upstream regulatory gene of ABCG1 in HCC.

### ABCG1 was contributed to oxaliplatin resistance, and inhibition of ABCG1 were one of the mechanisms of IP6 treatment on HCC oxaliplatin resistance

**Figure 5:**
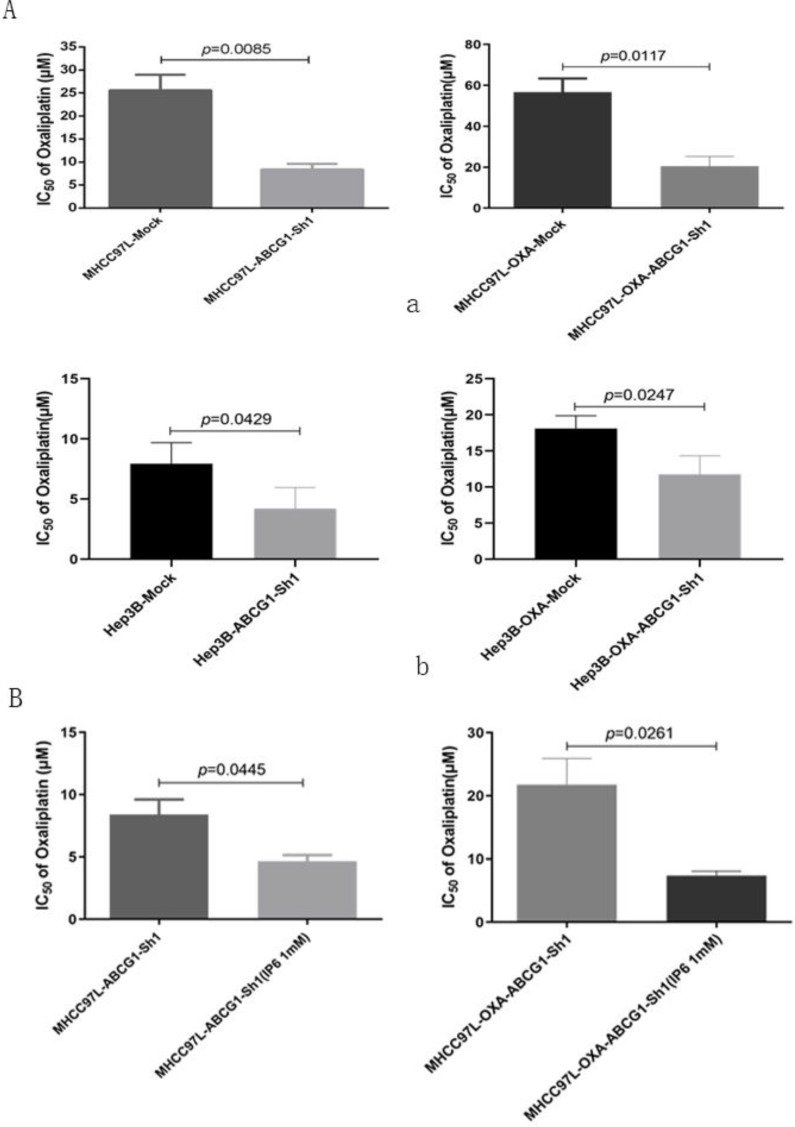
** Inhibition of ABCG1 reversed oxaliplatin resistance in HCC cells. (A)** Oxaliplatin IC50 values decreased both in MHCC97L-ABCG1-sh1 and MHCC97L-OXA-ABCG1-Sh1 compared to the corresponding mock cells, and the same trend was confirmed in Hep3B-ABCG1-sh1 and Hep3B-OXA-ABCG1-Sh1. **(B)** Oxaliplatin IC_50_ values still decreased in MHCC97L-OXA-ABCG1-Sh1 synergistically treated with IP6 compared to the control group.

ABCG1 was silenced using specific shRNA in MHCC97L and MHCC97L-OXA cells. Oxaliplatin IC_50_ values decreased in MHCC97L-ABCG1-sh1 compared to the mock cells (8.41 ± 2.09 μmol/L *vs.* 25.59 ± 5.82 μmol/L; *p* = 0.0085). Then, we also confirmed the decreased oxaliplatin IC_50_ in MHCC97L-OXA-ABCG1-Sh1 compared to the mock cells (20.47 ± 8.27 μmol/L* vs.* 56.67± 11.59 μmol/L; *p* = 0.0117; Figure 5A, a). Additionally, Oxaliplatin IC_50_ values also decreased in Hep3B-ABCG1-sh1 (4.21 ± 1.76 μmol/L *vs.* 7.93 ± 1.76 μmol/L; *p* = 0.0429) and Hep3B-OXA-ABCG1-Sh1 (11.77 ± 2.58 μmol/L *vs.* 18.11 ± 1.77 μmol/L; *p* = 0.0247; Figure 5A, b) cells compared with the associated mock cells.

Then we explored whether ABCG1 was the only factor affecting oxaliplatin resistance. ABCG1 was knocked down in MHCC97L and MHCC97L-OXA cells, and which were synergistically treated with IP6. We confirmed the decreased oxaliplatin IC_50_ in MHCC97L-ABCG1-sh1 synergistically treated with IP6 compared to the control group (4.66 ± 0.81 μmol/L* vs* 8.40± 2.07 μmol/L; *p* = 0.0445). Additionally, Oxaliplatin IC_50_ values also decreased in MHCC97L-OXA-ABCG1-Sh1 synergistically treated with IP6 compared to the control group (20.47 ± 8.27 μmol/L* vs* 56.67±11.59 μmol/L; *p* = 0.0261; Figure 5B). Together, these findings suggested that ABCG1 that contributed to oxaliplatin resistance, and inhibition of ABCG1 was one of the mechanisms of IP6 treatment on HCC.

## Discussion

For HCC patients with advanced stage disease, transcatheter arterial chemoembolization (TACE) and systemic chemotherapy are usually the treatments of choice 16. Although oxaliplatin is widely used in the treatment of advanced malignancies, its long-term treatment outcomes are far from being satisfactory, due to the presence of both intrinsic and acquired resistance. Previously, our group showed that residual HCC in oxaliplatin treatment underwent EMT, acquired increased oxaliplatin resistance and demonstrated increased pulmonary metastatic potential 17. Therefore, methods to enhance oxaliplatin treatment responses are urgently needed.

The consumption of whole grains and whole grain products has gained popularity because of potential protection against cancer 18. Whole grain phytochemicals regulate cellular signal transduction pathways and hence affect cancer cell behaviors such as proliferation, apoptosis, and invasion 19. IP6 is a naturally occurring polyphosphorylated carbohydrate that is abundant in grains and is known to act as a broad-spectrum anti-neoplastic agent. Its potential role as a combination therapy agent with oxaliplatin to date has been unexplored. In the present study, IP6 not only inhibited proliferation and migration in HCC cells, demonstrating individual value in the treatment of malignancies as shown in previous papers 20-22, but IP6 also exhibited a synergistic anti-proliferative effect when used in combination with oxaliplatin. HCC cells pre-treated continually with increasing concentration of IP6 also exhibited lower proliferation rates with enhanced chemosensitivity to oxaliplatin. All these findings suggest that IP6 reversed the tolerance of oxaliplatin-resistant HCC cells with a synergistic anti-proliferative effect.

ABCG1 is an ATP-binding cassette transporter and has a major role in inducing cellular cholesterol efflux to high-density lipoprotein (HDL) particles. ABCG1 deficiency in macrophages and smooth muscle cells leads to reduced cholesterol efflux and apoptosis 11, 23. It was also demonstrated that ABCG1 promotes the migration and invasion as a potential oncogene and therapeutic target in tumor cells 11, 24. We showed here that ABCG1 upregulation was involved in oxaliplatin resistance. Silencing ABCG1 using specific shRNA in HCC cells resulted in decreased oxaliplatin IC_50_ values, significantly reversing the oxaliplatin resistance. Our data also suggested ABCG1 was a downstream molecule of the CCN2-LRP6-Wnt/β-catenin signaling pathway in HCC. IP6, originating as a natural ingredient of whole grains, inhibited CCN2-Wnt/β-catenin signaling and downregulated ABCG1 expression levels in HCC cells, and therefore indicating CCN2-LRP6-Wnt/β-catenin-ABCG1 signaling was one of the mechanisms of IP6 treatment in HCC.

Evidence has shown that whole grains may protect against various cancers, and IP6 is a naturally occurring polyphosphorylated carbohydrate that is abundant in grains. In the present study, we demonstrated that combined chemotherapy with oxaliplatin and IP6 synergistically induced sensitizing effects in oxaliplatin-resistant HCC cells, providing a good alternative for combination or sequential therapy using IP6 for treatment of HCC.

## Conclusions

From our experimental results and our review of the literature, we propose the following conclusions. (1) IP6 exhibited a synergistic and sequential anti-proliferative effect with oxaliplatin in HCC. (2) ABCG1 was associated with oxaliplatin resistance and was acting as a downstream molecule of the CCN2-Wnt/β-catenin signaling pathway. (3) HCC cells treated with IP6 exhibited downregulation of ABCG1 and the CCN2-LRP6-β-catenin-ABCG1 signaling pathway. We conclude that IP6 exhibited a synergistic and sequential anti-proliferative effect with oxaliplatin in HCC.

## Abbreviations

HCC: hepatocellular carcinoma
IP6: Inositol hexaphosphate
EMT: epithelial-mesenchymal transition
DMEM: Dulbecco's Modified Eagle's Medium
FBS: fetal bovine serum
PCNA: proliferating cell nuclear antigen
CCK8: Cell Counting Kit 8
TACE: transcatheter arterial chemoembolization
HDL: high-density lipoprotein
LRP6: Low-density lipoprotein receptor-related protein-6
